# Superior Dispersal Ability Can Lead to Persistent Ecological Dominance throughout Succession

**DOI:** 10.1128/AEM.02421-18

**Published:** 2019-03-06

**Authors:** Primrose J. Boynton, Celeste N. Peterson, Anne Pringle

**Affiliations:** a Environmental Genomics Working Group, Max-Planck Institute for Evolutionary Biology, Plön, Germany; b Department of Biology, Suffolk University, Boston, Massachusetts, USA; c Departments of Botany and Bacteriology, University of Wisconsin—Madison, Madison, Wisconsin, USA; Kyoto University

**Keywords:** competition, dispersal limitation, high-throughput sequencing, metacommunities, purple pitcher plant, succession, yeast

## Abstract

A large number of descriptive surveys have shown that microbial communities experience successional changes over time and that ecological dominance is common in the microbial world. However, direct evidence for the ecological processes mediating succession or causing ecological dominance remains rare. Different dispersal abilities among species may be a key mechanism. We surveyed fungal diversity within a metacommunity of pitchers of the model carnivorous plant Sarracenia purpurea and discovered that the yeast Candida pseudoglaebosa was ecologically dominant. Its frequency in the metacommunity increased during the growing season, and it was not replaced by other taxa. We next measured its competitive ability in a manipulative laboratory experiment and tracked its dispersal over time in nature. Despite its dominance, C. pseudoglaebosa is not a superior competitor. Instead, it is a superior disperser: it arrives in pitchers earlier, and disperses into more pitchers, than other fungi. Differential dispersal across the spatially structured metacommunity of individual pitchers emerges as a key driver of the continuous dominance of C. pseudoglaebosa during succession.

**IMPORTANCE** Microbial communities are ubiquitous and occupy nearly every imaginable habitat and resource, including human-influenced habitats (e.g., fermenting food and hospital surfaces) and habitats with little human influence (e.g., aquatic communities living in carnivorous plant pitchers). We studied yeast communities living in pitchers of the carnivorous purple pitcher plant to understand how and why microbial communities change over time. We found that dispersal ability is not only important for fungal communities early in their existence, it can also determine which species is dominant (here, the yeast Candida pseudoglaebosa) long after the species and its competitors have arrived. These results contrast with observations from many human-influenced habitats, in which a good competitor eventually outcompetes good dispersers, since humans often design these habitats to favor a specific competitor. This study will help microbiologists understand the qualities of microbial species that enable takeover of new habitats in both natural and human-influenced environments.

## Abstract (precis)

Microbial communities are ubiquitous and occupy nearly every imaginable habitat and resource, including human-influenced habitats (e.g., fermenting food and hospital surfaces) and habitats with little human influence (e.g., aquatic communities living in carnivorous plant pitchers). We studied yeast communities living in pitchers of the carnivorous purple pitcher plant to understand how and why microbial communities change over time. We found that dispersal ability is not only important for fungal communities early in their existence, it can also determine which species is dominant (here, the yeast Candida pseudoglaebosa) long after the species and its competitors have arrived. These results contrast with observations from many human-influenced habitats, in which a good competitor eventually outcompetes good dispersers, since humans often design these habitats to favor a specific competitor. This study will help microbiologists understand the qualities of microbial species that enable takeover of new habitats in both natural and human-influenced environments.

## INTRODUCTION

Primary microbial succession occurs when a microbial community colonizes and develops on a newly available substrate (1). The advent of high-throughput sequencing has revolutionized observational studies of microbial succession, enabling researchers to describe the development of microbial communities in fine detail (2–5). A variety of successional patterns have been observed. For example, taxon diversity can increase, decrease, or randomly vary with successional time (4, 6, 7). However, researchers commonly observe replacement of early-successional taxa by late-successional taxa (2, 8). An ongoing challenge in microbial ecology is to connect observed ecological patterns to the ecological processes responsible for the patterns.

The development of ecological dominance by one or a few microbes over successional time is a particularly intriguing phenomenon (2, 9, 10), in part because of its obvious parallels to plant and animal ecology (11). Ecological dominance is apparent when one or a few species comprise most of the individuals or biomass in a community (11). Environmental filtering, superior competitive ability of the dominant species, and ecosystem engineering can all cause dominance (8, 12–15). For example, Saccharomyces cerevisiae dominance in wine results from environmental changes, caused by S. cerevisiae itself, that make the fermentation environment hospitable to S. cerevisiae and inhospitable to other microbes (13, 16). As another example, algal dominance during bloom events can be the result of environmental filtering caused by eutrophication (17). However, the dynamics of microbial dominance have been primarily studied in domesticated systems, and the most frequently reported mechanisms may not be responsible for dominance in all, or even most, natural microbial communities. For example, environmental filtering and competition may be more important in systems where human beings have designed environments to favor domesticated microbes (18) and less important in natural environments with heterogeneous environmental conditions.

An overlooked mechanism driving ecological dominance in natural systems may be dispersal (i.e., arrival in new habitats). When primary succession occurs on a sterile substrate, all members of the microbial community must first disperse onto the new substrate before establishing in the community. A good disperser may prevent the establishment of other community members through priority effects if it arrives in a habitat first, either by preempting or modifying available niches (19). In addition, good dispersers in nearby communities can impact a microbial community during succession by producing propagules that then disperse into the developing community (20).

Dispersal may emerge as a key driver of ecological dominance in microbial metacommunities. Metacommunities are physically structured groups of communities in which individual communities are spatially isolated from one another but linked through dispersal (21, 22). Community assembly in metacommunities is a function of dispersal among communities and ecological processes occurring inside each community. Ecological theory explains how dispersal can interact with intracommunity processes to maintain metacommunity diversity (21, 23). For example, populations occupying low-quality environmental patches can be maintained by dispersal from high-quality patches (“mass effects”), or fitness trade-offs between competitive ability and dispersal ability can mediate species diversity (“patch dynamics”). Theory predicts that dispersal and competition interact during succession in the individual component communities of a metacommunity and the result is a hump-shaped relationship between successional time and species richness (24, 25). Species richness is predicted to be low early in a component community’s age, to increase as more species disperse into the community, and then to decrease as competitive interactions remove species from the community. Although rarely documented, it is also possible that a particularly good disperser will become dominant and remain dominant (and therefore decrease community diversity) in a metacommunity solely as a result of its dispersal ability.

**FIG 1:**
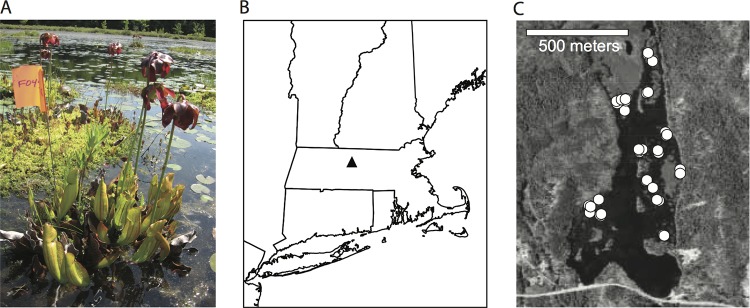
Example S. purpurea plant and study location. (A) One of the study pitcher plants at the edge of a *Sphagnum* island. This photograph was taken early in the growth season and both opened and unopened pitchers are visible. (B) Location of Harvard Pond in Massachusetts. (C) Locations of the 43 pitchers sampled for this study. Each white circle represents one pitcher. Note that some pitchers were close enough to one another that the white circles overlap. Maps were created using ArcMap version 9.2 (77); map data are from previous publications (78, 79).

We investigated the contribution of dispersal to ecological dominance over the course of natural fungal succession in pitchers of the carnivorous pitcher plant Sarracenia purpurea. S. purpurea is a perennial plant native to bogs and savannas in northern and eastern North America (26). Each S. purpurea plant produces modified vase-shaped leaves, or pitchers, annually (Fig. 1A). At first, developing pitchers are entirely closed, sterile chambers (27). Once mature, the top portion of each pitcher opens, and the open pitchers accumulate rainwater to form small pools of water (phytotelmata). Potential prey (ants and other small insects) are attracted to pitchers (28); some prey fall into pitchers and drown and are then shredded, decomposed, and mineralized by a food web of microorganisms and invertebrates (29). The pitcher microbial community includes bacteria, algae, and fungi, including culturable yeasts (30). Yeasts have long been recognized as components of pitcher plant food webs, and diverse fungi are readily detected by sequencing when eukaryote-specific primers are used (30, 31). Pitchers are individual communities within a metacommunity of other pitchers on the same and on different plants.

We followed fungal succession within individual pitchers in a *Sphagnum* bog in central Massachusetts, where the pitcher growing season lasts for about three months (although pitchers persist for longer and can overwinter) (32). We first documented that a single yeast species, Candida pseudoglaebosa, was numerically dominant in the fungal metacommunity throughout the growing season, and its frequency increased between early- and late-successional stages of individual pitchers. We next investigated the ecological processes leading to C. pseudoglaebosa dominance. Unlike dominant yeasts in many other systems, C. pseudoglaebosa was not an especially good competitor against other tested yeasts. However, it was an especially good disperser. In the pitcher plant metacommunity, dispersal is defined as an organism’s arrival or appearance in a pitcher previously unoccupied by that species. C. pseudoglaebosa is one of the first fungi to arrive in pitchers, and it maintained high frequency in the pitcher plant metacommunity over the course of the season, even as its frequency within individual pitchers ranged from completely absent to more than 90% of the sequences. The apparently superior dispersal ability of C. pseudoglaebosa leads it to dominate the pitcher fungal metacommunity, demonstrating that dispersal ability, like competitive ability, is an important contributor to ecological dominance in microbial communities.

## RESULTS

### Fungal communities in pitchers change over successional time.

To understand how fungal communities develop in pitchers, we first sequenced entire fungal communities in seventeen pitchers over the course of a growing season. Before selecting pitchers to track for sequencing, we identified 43 unopened pitchers on *Sphagnum* islands in Harvard Pond, located in Petersham, Massachusetts (Fig. 1). We recorded the opening date of each pitcher and collected water from each pitcher at 4 days, 7 days (“1 week”), 34 to 42 days (“1 month”), and 66 to 74 days (“2 months”) after opening. At the 2-month time point, insect herbivores, including moth larvae (33), had destroyed 10 of the original 43 pitchers, and we could only sample water from 33 pitchers. In the sampled pitchers, the presence of fungal DNA was assayed using the ITS1F/ITS4 primer pair (34, 35). Fungi were detectable starting from the first measured time point at 4 days (in 33% of sampled plants), and were widespread after 1 week, 1 month, and 2 months (in 88, 95, and 73% of the sampled plants, respectively). Fungi were detected in 100% of sampled pitchers at least once during the season, and 17 of the pitchers contained detectable fungal DNA at every time point from 1 week to 2 months. We sequenced fungal DNA from these seventeen pitchers at all available time points, including 4 days if available, using 454 sequencing of PCR amplicons of the internal transcribed spacer (ITS) ribosomal region. We chose to sequence the ITS region because it is a common barcode used to discern fungal species (36). While this region is generally able to distinguish between species, it is not useful for measuring intraspecific genetic diversity.

**FIG 2:**
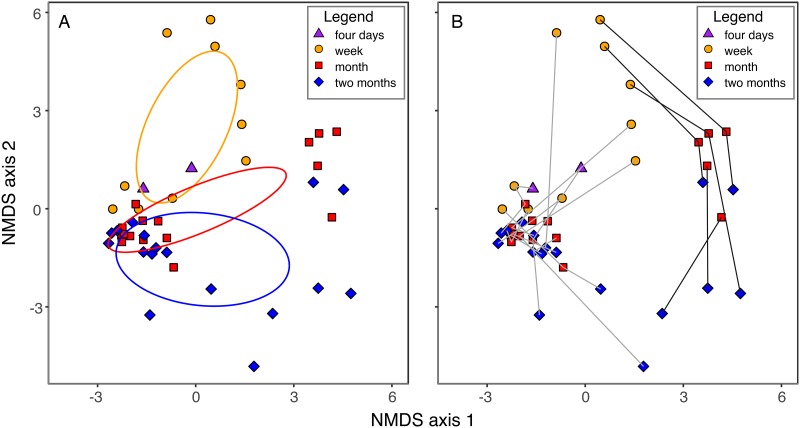
Nonmetric multidimensional scaling (NMDS) plots of pitcher plant community similarities. Similarities of community OTU compositions were calculated using the Jaccard metric (70). Purple triangles represent 4-day-old communities, orange circles represent week-old communities, red squares represent approximately 1-month-old communities, and blue diamonds represent approximately 2-month-old communities. (A) NMDS plot with similarities among time points highlighted. Ellipses depict 95% confidence intervals of the centroid of each time point. No ellipse is depicted for 4-day-old communities because only two communities were measured. (B) NMDS plot with individual pitchers highlighted. Lines connect measurements for each pitcher. Fungal communities in pitcher group 1 are represented with black lines and fungal communities in pitcher group 2 are represented with gray lines. All points are located at the same coordinates in both panels.

Fungal succession varied among pitchers. While community composition changed significantly with time (Fig. 2A), only a small amount of variation in community composition was due to variation in time (distance-based redundancy analysis adjusted *R*^2^ = 0.03, F = 2.00, df = 1,27, *P* = 0.012). This small influence of time on community composition was likely a result of high variation among pitchers. Succession followed two trajectories—five pitchers (here referred to as “pitcher group 1”) followed a different successional trajectory from the other twelve (“pitcher group 2”)—and there was considerable variation among communities within each trajectory (Fig. 2B). Distance among pitchers did not explain significant variation in community composition (partial mantel test of community composition on space controlling for time, mantel statistic *r* = −0.03, significance = 0.696).

**FIG 3:**
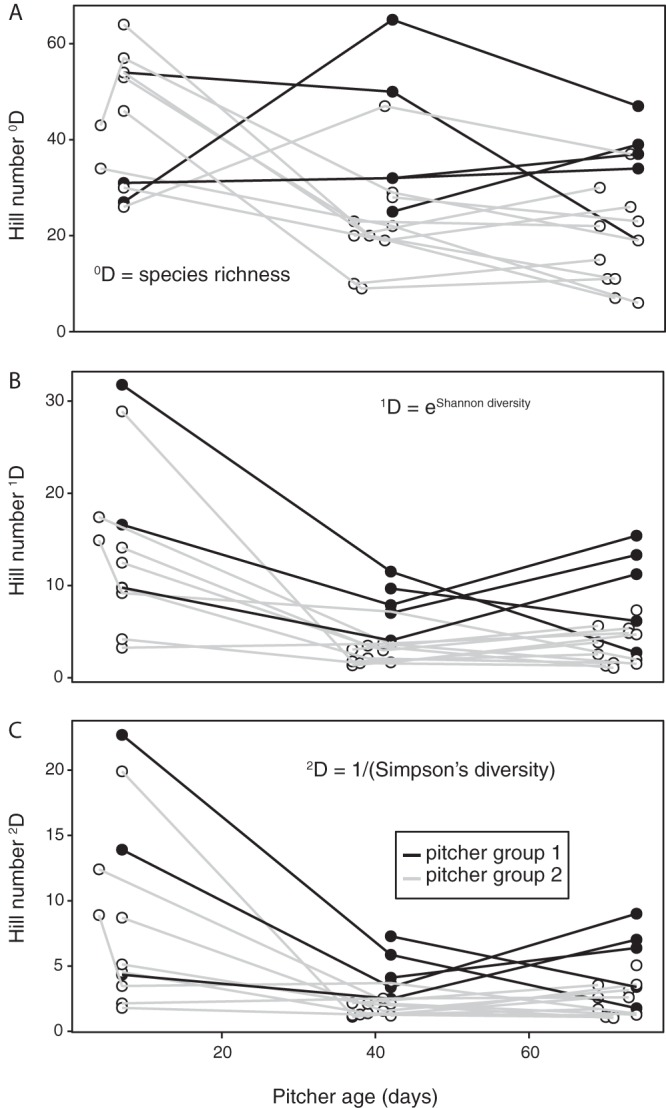
Hill numbers of orders 0 (A), 1 (B), and 2 (C) in pitchers over time. Data points for communities in the same pitcher are connected with lines. Black lines connect points for pitcher group 1, and gray lines connect points for pitcher group 2.

Despite the observed variation in fungal community composition, diversity decreased, on average, in pitchers between 4 days and 2 months (Fig. 3). To determine a sample’s diversity, we calculated Hill numbers of orders 0 to 2 (^0^D to ^2^D) for each sample after rarefaction to 1,143 sequences per sample. Hill numbers of different orders give community diversity with an emphasis on rare species (low orders) or common species (high orders) (37, 38). ^0^D, ^1^D, and ^2^D are equal to operational taxonomic unit (OTU) richness, the exponent of Shannon diversity, and the inverse of Simpson’s index, respectively. Diversity as indicated by all calculated Hill numbers decreased between early and late time points: on average, ^0^D declined significantly from 43.3 within the first week (including 4-day and 1-week time points) to 23.9 after 2 months (*t* = –3.5, df = 27, *P* = 0.002); ^1^D declined from 14.4 to 5.3 (*t* = –3.6, df = 27, *P* = 0.001); and ^2^D declined from 9.0 to 3.3 (*t* = –3.2, df = 27, *P* = 0.004).

### *C. pseudoglaebosa* is the dominant fungal taxon in pitchers throughout succession.

**FIG 4:**
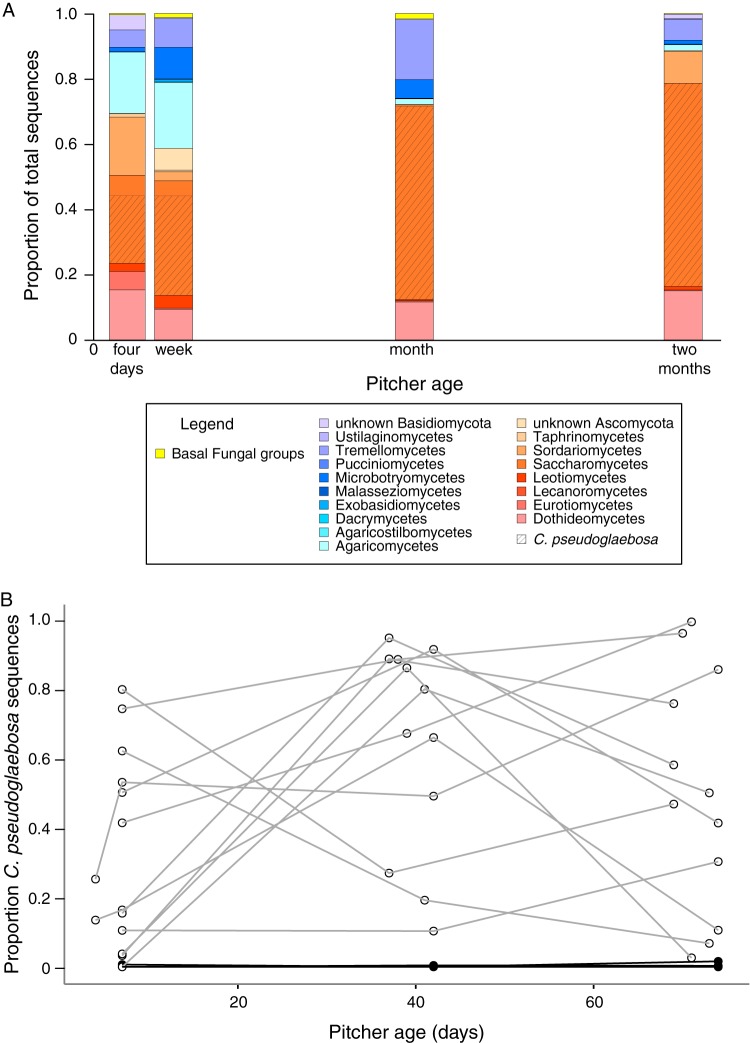
Taxon diversity in pitchers over time. Proportions are reported based on nonrarefied OTU assignments. (A) Taxon diversity in the entire bog metacommunity. Colored bars represent proportions of total sequences for each fungal class (or phylum for basal fungal lineages). Unclassified sequences were not included in this figure. The hatched area represents total C. pseudoglaebosa frequency for each time point. Note that C. pseudoglaebosa is in the class *Saccharomycetes* and represents over 99% of *Saccharomycetes* sequences at the 1- and 2-month time points. (B) C. pseudoglaebosa sequence frequency in individual pitcher communities. Data points for communities in the same pitcher are connected with lines. Black lines connect points for pitcher group 1, and gray lines connect points for pitcher group 2.

C. pseudoglaebosa, in the class *Saccharomycetes*, was the numerically dominant taxon in the fungal metacommunity, but was not dominant in every pitcher (Fig. 4). In the metacommunity, C. pseudoglaebosa was more frequent than any other taxon at every time point and its frequency increased between early- and late-successional time points. Its metacommunity frequency increased from 19% of total sequences at 4 days to 42% at 2 months (Fig. 4A). However, its frequency did not increase over time in every pitcher. Depending on the pitcher, C. pseudoglaebosa’s within-pitcher frequency increased over the season, decreased over the season, peaked midway through the season, or dipped midway through the season (Fig. 4B). We cannot say whether these increases or decreases in C. pseudoglaebosa sequence frequency reflect changes in the total cell numbers because we did not measure cell numbers or fungal biomass in pitchers. Pitcher group 1 never contained appreciable C. pseudoglaebosa: each pitcher in group 1 contained less than 1.6% C. pseudoglaebosa sequences regardless of the sampled time point (Fig. 4B).

### *C. pseudoglaebosa* is not a superior competitor, but it has complex interactions with other yeasts.

To better understand how interactions with other yeasts might influence the frequency of C. pseudoglaebosa, we grew C. pseudoglaebosa and other potentially interacting pitcher yeasts in laboratory microcosms. We followed the strategy advocated in (39), which suggested determining interacting species’ effects on one another by measuring organism performance as the number of interacting individuals increases. We inoculated microcosms with all possible pairs of three culturable pitcher yeasts (C. pseudoglaebosa, Rhodotorula babjevae, and Moesziomyces aphidis). C. pseudoglaebosa represented 41%, R. babjevae represented 2%, and M. aphidis represented 0.06% of total sequences in the sequencing data set. Each low-nutrient microcosm contained a focal yeast, which was inoculated as a fixed number of cells, and an interactor, which was inoculated as a varying number of cells. We then let the pairs of yeasts grow in the microcosms and investigated the effects of interactors on each focal yeast using regressions. We evaluated interaction qualities based on the direction (increasing or decreasing focal yeast yield with more interacting cells) and shape (linear or polynomial) of each regression, and we evaluated differences between interactor yeasts based on whether adding interactor yeast identity to each regression improved its fit.

**FIG 5:**
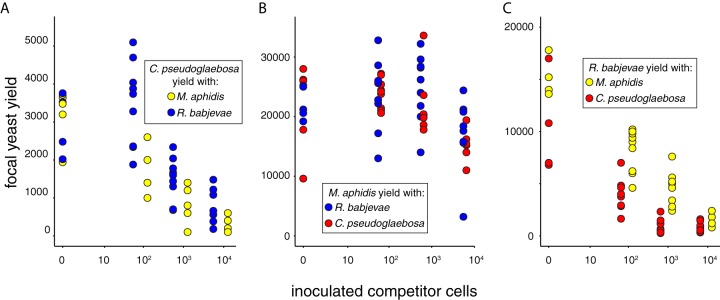
Influence of interacting species on C. pseudoglaebosa (A), M. aphidis (B), and R. babjevae (C). The plots depict the yield of each focal species as a function of the number of cells of an interacting species coinoculated with the focal species. Interacting species are coded by color: red, C. pseudoglaebosa; yellow, M. aphidis; and blue, R. babjevae.

Interactions between pitcher plant yeasts ranged from facilitation to competition, depending on the identities of the yeasts and the number of interactor cells present. Under microcosm conditions, interactions between focal yeasts and interactors were polynomial when the focal yeast was C. pseudoglaebosa or M. aphidis (Fig. 5A and B, Tables 1 and 2): both yeasts were facilitated by small numbers of coinoculated cells, but their growth was impeded by larger numbers of coinoculated cells. Note that we detected facilitation of C. pseudoglaebosa by R. babjevae when few R. babjevae cells were inoculated, but we did not inoculate M. aphidis in small enough numbers to confirm M. aphidis facilitation of C. pseudoglaebosa (Fig. 5A). At high numbers of coinoculated cells, M. aphidis had a more detrimental impact on C. pseudoglaebosa than R. babjevae (F = 6.79, df = 1,50, *P* = 0.012, Fig. 5, Table 2). In contrast, the two interactors of M. aphidis had similar effects on its yield: at low and intermediate inoculum sizes, both R. babjevae and C. pseudoglaebosa promoted M. aphidis growth, but at high inoculum sizes, both interactors inhibited M. aphidis growth (Fig. 5B). Interactions between R. babjevae and interactor yeasts were linear (Fig. 5C, Tables 1 and 2): R. babjevae yield was impeded by interactors regardless of the number of interactor cells present, and C. pseudoglaebosa had a more detrimental impact on R. babjevae than M. aphidis did (F = 86.07, df = 1,58, *P* < 0.001, Table 2). In addition, microcosms with larger interactor inoculum sizes produced more interactor cells at the end of the experiment. In other words, the more of a species’ cells we inoculated at the start of the experiment, the more cells we counted at the end of the experiment, regardless of whether the species was a focal or interactor species. The observation that C. pseudoglaebosa and R. babjevae are inhibited, whereas M. aphidis is facilitated, by intermediate numbers of competing cells was qualitatively supported by data from a three-way competition test, in which microcosms were inoculated with approximately 1,000 cells of each species. In these microcosms, M. aphidis increased in frequency from 51 to 88% of all cells, while C. pseudoglaebosa and R. babjevae each decreased, from 26 and 22% to 4 and 8% of all cells, respectively.

**TABLE 1:** Model selection for each focal yeast^*a*^

Response variable and explanatory variables^*b*^	df	F	*P*	Adjusted *R*^2^	AIC
No. of C. pseudoglaebosa cells					
Inoculum size	1, 51	79.37	<0.001	0.60	405.64
(Inoculum size)^2^	1, 51	111.1	<0.001	0.68	394.09
Inoculum size + (inoculum size)^2^	2, 50	54.65	<0.001	0.67	395.97
**(Inoculum size)^2^ + competitor identity**	**2, 50**	**65.26**	**<0.001**	**0.71**	**389.34**
(Inoculum size)^2^ + competitor identity × (inoculum size)^2^	2, 50	60.02	<0.001	0.69	392.51
(Inoculum size)^2^ + competitor identity + competitor identity × (inoculum size)^2^	3, 49	42.74	<0.001	0.71	391.25
					
No. of M. aphidis cells					
Inoculum size	1, 58	5.37	0.02	0.07	1,211.77
(Inoculum size)^2^	1, 58	11.97	0.001	0.16	1,205.83
**Inoculum size + (inoculum size)^2^**	**2, 57**	**10.86**	**<0.001**	**0.25**	**1,199.71**
Inoculum size + (inoculum size)^2^ + competitor identity	3, 56	7.64	<0.001	0.25	1200.51
Inoculum size + (inoculum size)^2^ + competitor identity × inoculum size	3, 56	7.73	<0.001	0.26	1,200.28
Inoculum size + (inoculum size)^2^ + competitor identity × (inoculum size)^2^	3, 56	7.68	<0.001	0.25	1,200.28
					
No. of R. babjevae cells					
Inoculum size	1, 59	92.25	<0.001	0.60	542.62
(Inoculum size)^2^	1, 59	67.64	<0.001	0.53	553.45
Inoculum size + (inoculum size)^2^	2, 58	45.56	<0.001	0.60	544.44
**Inoculum size + competitor identity**	**2, 58**	**155.7**	**<0.001**	**0.84**	**489.12**
Inoculum size + competitor identity × inoculum size	2, 58	109.0	<0.001	0.78	506.87
Inoculum size + competitor identity + competitor identity × inoculum size	3, 57	102.1	<0.001	0.83	491.06

a Information in bold indicates the best-fitting model for each focal yeast.

b The inoculum size was log_10_(*x* + 1) transformed. Numbers of C. pseudoglaebosa and R. babjevae cells were square-root transformed.

**TABLE 2:** ANOVA data for each best-fitting model

Response variable and explanatory variable^*a*^	df	SS	MS	F	*P*
No. of C. pseudoglaebosa cells					
(Inoculum size)^2^	1	10,235.2	10,235.2	123.73	<0.001
Competitor identity	1	561.4	561.4	6.79	0.012
Residuals	50	4,136.3	82.7		
					
No. of M. aphidis cells					
Inoculum size	1	173,667,504	173,667,504	6.6684	0.012
(Inoculum size)^2^	1	391,953,317	391,953,317	15.0501	<0.001
Residuals	57	1,484,466,512	26,043,272		
					
No. of R. babjevae cells					
Inoculum size	1	36,941	36,941	225.28	<0.001
Competitor identity	1	14,114	14,114	86.07	<0.001
Residuals	58	9,511	164		

a The inoculum size was log_10_(*x* + 1) transformed. Numbers of C. pseudoglaebosa and R. babjevae cells were square-root transformed.

### *C. pseudoglaebosa* is an early disperser in pitchers.

**FIG 6:**
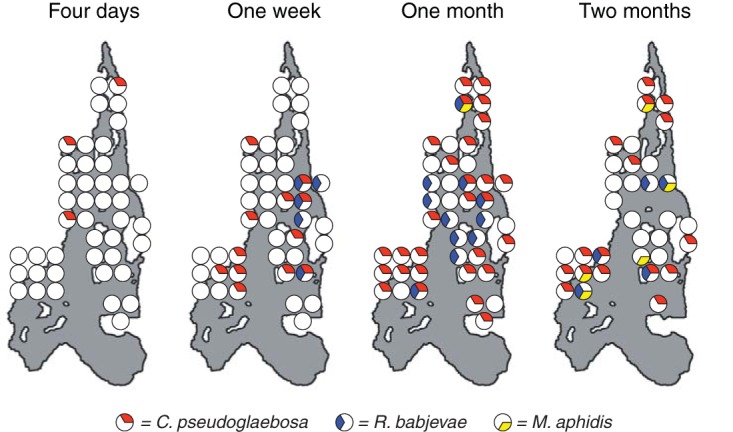
Presences and absences of each of three yeasts in 43 pitchers over time. Each large gray shape represents the Harvard Pond at one of four time points. Circles represent pitchers: completely white circles represent pitchers in which none of the three yeasts were detected, and circles containing colored pie slices represent pitchers in which one or more of the three assayed yeasts were detected. Pie slices are colored by detected yeast: red, C. pseudoglaebosa; yellow, M. aphidis; and blue, R. babjevae. Circles indicate the approximate locations of pitchers, and are offset to make all data visible; see Fig. 1C for accurate pitcher locations. Map data are from reference 78.

To investigate whether dispersal influences C. pseudoglaebosa dominance in pitchers, we observed the arrival times each of the three yeasts mentioned above in pitchers over the S. purpurea growth season in Harvard Pond. We surveyed the presence or absence of each yeast in each of the 43 sampled pitchers using taxon-specific PCR primers (Table 3) to determine when each yeast arrived in a pitcher and whether it persisted throughout the season. We sought to amplify DNA of each of the three species from all samples, including those in which fungi had not previously been detected using the more general ITS1F/ITS4 primer pair. The three yeasts appeared in pitchers sequentially (Fig. 6): C. pseudoglaebosa first arrived in pitchers within 4 days after the pitchers opened, R. babjevae arrived between 4 days and 1 week after pitchers opened, and M. aphidis arrived 1 week to 1 month after pitchers opened. Once a yeast colonized a pitcher, it either persisted in or disappeared from that pitcher later in the season, but it did not disappear from the broader metacommunity.

**TABLE 3:** Taxon-specific PCR primer sequences used to detect pitcher yeasts

Yeast	Forward primer		Reverse primer		Product length (bp)
	Sequence	*T_m_*(ºC)	Sequence	*T_m_*(ºC)	
C. pseudoglaebosa	CTGCGGAAGGATCATTACAGT	54.6	TGTTCAGACAACACTGTTCA	51.8	466
R. babjevae	AAGTCGTAACAAGGTTTCCG	52.8	CCCAACTCGGCTCTAGTAA	53.9	527
M. aphidis	GGTAATGCGGTCGTCTAAAA	52.6	CTCTTCCAAAGAAGCGAGG	53.1	467

## DISCUSSION

### *C. pseudoglaebosa* is dominant in the pitcher metacommunity.

C. pseudoglaebosa was the dominant fungal taxon throughout succession in the S. purpurea pitcher metacommunity, although it did not dominate every individual pitcher community. It was the most frequent fungal taxon in the metacommunity at every sampled time point, and its frequency in the metacommunity increased after initial colonization (Fig. 4). The observation that C. pseudoglaebosa sequences were the most frequently found in the metacommunity is consistent with observations that C. pseudoglaebosa is the most readily cultured yeast taxon in pitchers and that it is as geographically widespread as the pitcher plant S. purpurea itself, found at sites spanning Florida, Newfoundland, and British Columbia (40). Dominance in the pitcher metacommunity was in part a result of the metacommunity structure itself: while C. pseudoglaebosa was not dominant in every pitcher, it was dominant in enough pitchers (in some cases with a frequency above 90% of all OTUs) to dominate the metacommunity as a whole (Fig. 4). The poor competitive performance of C. pseudoglaebosa relative to other yeasts in microcosms suggests that overall competitive superiority was not the cause of its dominance (Fig. 5). Instead, C. pseudoglaebosa’s early dispersal is a more likely cause (Fig. 6). Dispersal ability appears to enable C. pseudoglaebosa to maintain dominance in the metacommunity even when it is not dominant inside every pitcher it colonizes.

Our best explanation for C. pseudoglaebosa’s dominance is that it preempts other community members by reaching and establishing in pitchers before other taxa arrive. Early dispersal likely gives C. pseudoglaebosa a numerical advantage by providing the opportunity to begin exponential growth before other members of the fungal community can arrive and begin growing (41). In addition, once C. pseudoglaebosa is established in a pitcher, facilitation by low-frequency interacting taxa may help it to maintain dominance (Fig. 5A). Once established in the metacommunity, C. pseudoglaebosa will continue to disperse into new pitchers throughout the growing season (Fig. 6).

However, we do not understand how yeasts, including C. pseudoglaebosa, disperse into pitchers. Pitchers are sterile before they open, and all pitcher-inhabiting organisms must reach the pitcher habitat from the external environment (27). Because yeasts are ubiquitous in nature (42), potential sources of yeast inocula are numerous and include rainfall, older pitchers, the surfaces of other bog plants, surface or pore water of the bog itself, and surrounding forests and upland soils. It is also likely that pitcher invertebrates bring yeasts into pitchers, perhaps from older pitchers. C. pseudoglaebosa, or a close relative, was previously found associating with the pitcher-endemic mosquito W. smithii, and could persist in the hindguts of the mosquito Aedes aegypti in a laboratory experiment (43). However, we have no direct observations of insects introducing yeasts into sterile pitchers.

### Ecological patterns within pitchers.

Although C. pseudoglaebosa was the numerically dominant fungal taxon in the metacommunity of pitchers, chance events, dispersal, and interactions among fungi appear to determine whether it is the dominant taxon inside any given pitcher. We observed both facilitation and inhibition in microcosms (Fig. 5). While we did not investigate the mechanisms driving these interactions, inhibition may result from differences in abilities to exploit resources or from direct antagonism (44). We further hypothesize that interactions with the taxa that arrive, by chance, at different times in different pitchers caused the variety of C. pseudoglaebosa relative frequency changes observed (i.e., increasing, decreasing, or nonmonotonic, Fig. 4B) because different interacting yeasts have qualitatively different impacts on C. pseudoglaebosa depending on the number of interacting cells present (Fig. 5A). In general, individual pitchers experienced priority effects: the timing of taxon arrival in each pitcher (e.g., early arrival of C. pseudoglaebosa in a pitcher) determined later community composition (e.g., C. pseudoglaebosa dominance in the pitcher) (19). Despite the influence of chance events on C. pseudoglaebosa dominance in any given pitcher, the early and frequent dispersal of C. pseudoglaebosa compared to other yeasts enabled its overall dominance in the metacommunity (Fig. 4A and 6).

A variety of other ecological processes may influence C. pseudoglaebosa frequency changes in individual pitchers (Fig. 4B). In addition to interacting with other yeast species, C. pseudoglaebosa most likely interacts with bacteria. Bacteria might influence C. pseudoglaebosa dominance by altering pitcher environment pH or making nutrients available to C. pseudoglaebosa, or C. pseudoglaebosa might compete with bacteria for nutrients. Similarly, invertebrates in the pitcher food web community (29) likely influence C. pseudoglaebosa frequency, perhaps through predation.

Interactions between fungi and insect prey may also influence fungal communities and C. pseudoglaebosa frequencies in pitchers. S. purpurea pitchers generally trap most of their prey early in development, and this prey can be the only input into S. purpurea food webs (45). We observed an overall decline in fungal colonization late in succession (73% of pitchers with detectable fungi after two months, down from a high of 95%; note that only 33% of pitchers contained detectable fungi at the four day time point, probably because fungi had not yet had enough time to colonize sterile pitchers [27]). We attribute this decline to old pitchers experiencing die-offs of their fungal communities after all trapped prey were digested and nutrients were exhausted. In this way, pitchers resemble laboratory batch cultures, each of which has a limited amount of exhaustible nutrients. Variation in the quality and quantity of these exhaustible nutrients may also influence variation in C. pseudoglaebosa frequency among pitchers. Overall, while the superior dispersal ability of C. pseudoglaebosa allowed it to maintain its dominance across pitchers in the metacommunity, interactions with the pitcher host, other microbes, and insect prey were likely responsible for the fate of C. pseudoglaebosa in any given pitcher.

### Ecological patterns and processes during pitcher metacommunity succession.

In the metacommunity, and in many individual pitchers, C. pseudoglaebosa remained dominant through decreases in fungal taxon richness and diversity (Fig. 3 and 4). We did not observe a hump-shaped relationship between pitcher age and species richness, as previously predicted (Fig. 3A) (24). Species richness instead decreased, even as R. babjevae and M. aphidis were first dispersing into pitchers late in the season (Fig. 3A and 6). It is likely that C. pseudoglaebosa repression of taxa through priority effects has a larger influence on species richness than does new dispersal by other species, which would increase diversity.

Previous studies have also documented biotic and abiotic successional changes in pitchers; while we did not measure the same parameters as these previous studies, we assume that similar changes occurred in our pitcher metacommunity and that C. pseudoglaebosa maintained its dominance through these changes. For example, previous studies have documented decreasing pH with increasing pitcher age, an early peak in prey insect capture during pitchers’ life spans (46), and a variety of changes in bacterial, protist, and invertebrate community compositions over time (47, 48). In bacterial, protist, and invertebrate communities, the identities of dominant taxa changed as succession progressed. In contrast, C. pseudoglaebosa remained the dominant fungus throughout succession. C. pseudoglaebosa appears to be a classical early-successional taxon (1, 49) because it disperses early and frequently (Fig. 6), but it is not replaced by late-successional taxa.

Unlike classical early successional taxa, C. pseudoglaebosa maintains dominance in the metacommunity over time and is not consistently replaced. In contrast, dominant early successional taxa are replaced over time by dominant late-successional taxa in most studied successional systems (8, 46, 49, 50). Changes in the abiotic and biotic environment generally cause this turnover of dominant taxa (1, 49). In classical models of succession, early successional taxa are either superior dispersers, or are good at establishing in uncolonized habitats, or both. Late-successional taxa either require facilitation by early-successional taxa, or are tolerant of late-successional environmental conditions, or both (1, 49). Superior competitive ability and/or ecosystem engineering can also be responsible for late-successional dominance. For example, directly antagonistic interactions are often responsible for replacement of one species by another during fungal succession on decaying wood (51, 52). In contrast, C. pseudoglaebosa does not require environmental changes to achieve late-successional dominance, as it is already present and dominant early in succession, nor is it replaced by superior competitors late in succession.

C. pseudoglaebosa dominance throughout succession may be enabled by the short lifespans of pitchers in Harvard Pond; i.e., pitchers may not live long enough to enable late-succession fungal taxa to dominate the metacommunity. We sampled pitchers that were up to 66 to 74 days old, and stopped sampling at this age because 23% of pitchers had been destroyed by moths. However, pitchers in northern S. purpurea populations can survive intact through winter conditions (32), and pitchers can be active for over a year in the southern United States (48). We speculate that fungal succession more closely resembles classical successional patterns and the patterns observed for other pitcher guilds (e.g., bacteria, invertebrates) in longer-lived pitchers. For example, it is possible that a strong competitor such as M. aphidis could replace C. pseudoglaebosa in southern S. purpurea metacommunities where pitchers are active for many months. However, consistent dominance of a single taxon over succession may be common in microbial habitats that, like northern S. purpurea pitchers, have short lifespans but repeatedly become available.

### Conclusions.

In the model pitcher plant metacommunity, taxon dispersal ability has a profound influence on community structure. In particular, C. pseudoglaebosa’s ability to disperse into pitchers before other fungal taxa enables it to persist as the dominant taxon in the pitcher metacommunity, even as intertaxon interactions and the stochasticity of individual dispersal events prevent its dominance in every pitcher. It is likely that dispersal ability leads to persistent dominance in a variety of other natural succeeding microbial communities and metacommunities, especially when early dispersal allows a taxon to prevent establishment of other taxa. But to establish dispersal as a general mechanism causing dominance in microbial ecosystems, it would be useful to track its dynamics in other metacommunities.

Future studies of microbial succession should explicitly include metacommunity structure when investigating ecological processes. In the pitcher metacommunity, the overall taxon composition changed little over time, with C. pseudoglaebosa dominant throughout succession (Fig. 4A). However, individual pitchers followed a variety of trajectories (Fig. 2, 4B, and 6). Studies of succession that do not take a metacommunity’s structure into account may miss community heterogeneity and the diversity of ecological processes, especially dispersal ability, in play among communities.

## MATERIALS AND METHODS

### Study site and field collections.

Observations were made on *Sphagnum* islands in Harvard Pond, adjacent to Tom Swamp, a 50-ha *Sphagnum* bog located in Petersham, MA, at 42°30′N, 72°12′W (Fig. 1) (53). The C. pseudoglaebosa and M. aphidis isolates used in the microcosm study were collected from pitchers in Harvard Pond, and the R. babjevae isolate was collected from a pitcher in Swift River Bog, a 2-ha kettlehole bog located 75 km south of Tom Swamp in Belchertown, MA, at 42°16′N, 72°20′W (54). These three yeast isolates were collected in the summer of 2006 and identified by comparing their ribosomal sequences, amplified using the ITS1F/ITS4 and LS1/LR5 primer pairs (34, 35, 55, 56), to sequences in the NCBI BLAST database (57). We chose C. pseudoglaebosa, R. babjevae, and M. aphidis in part because they were all easily cultured from pitchers and in part because they formed colonies with different morphologies on agar plates: C. pseudoglaebosa forms smooth white colonies, M. aphidis forms wavy white colonies, and R. babjevae forms smooth pink colonies.

All S. purpurea pitcher water samples for PCR and 454 sequencing were collected in the spring and summer of 2009. In May of 2009, we identified 43 unopened S. purpurea pitchers on 32 *Sphagnum* islands in Harvard Pond. Pitchers ranged from less than 1 m to 908 m in distance to other pitchers (Fig. 1C). To the best of our knowledge, all pitchers were taken from different rosettes. However, rosettes can be joined via rhizomes hidden underwater or underground, and we did not confirm that all sampled pitchers were from genetically distinct plants. We visited each pitcher daily until it opened, and counted pitcher age from the date it opened. For each pitcher water collection, the water inside a pitcher was first mixed by pipetting up and down with a sterile plastic transfer pipette. We then removed about 0.25 ml of pitcher water and mixed it with 0.25 ml of CTAB buffer (100 mM Tris [pH 8.0], 1.4 M sodium chloride, 20 mM EDTA, 2% CTAB). To the best of our ability, we avoided collecting insect prey or macrofauna in these samples, although any protists and microscopic animals present in our samples were included; collected pitcher water contained no large animal parts and appeared as a cloudy liquid. All samples were flash-frozen in liquid nitrogen within 5 h of collection and stored at −20 or –80°C before DNA extraction.

### PCR assay.

We assayed each pitcher water sample for amplifiable DNA from all fungi, using the ITS1F/ITS4 primer pair, and for each of the three yeasts in the microcosm experiment, using the primers in Table 3. Primers to selectively amplify portions of each microcosm yeast’s ITS sequence were designed using the NCBI BLAST primer tool (58). We chose primer sequences to reliably amplify as much of the ITS sequence of each yeast species as possible, while not amplifying other sequences in the BLAST database.

To extract DNA from each pitcher water sample before the PCR assay, we first thawed and centrifuged frozen samples at 16,100 × *g* for 10 min and removed the supernatant from each pellet. We then suspended each pellet in 200 μl of breaking buffer (2% Triton X-100, 1% sodium dodecyl sulfate, 100 mM sodium chloride, 10 mM Tris, and 1 mM EDTA) (59). We mixed each suspension with about 200 μl of 0.5-mm glass beads and 200 μl of chloroform-phenol-isoamyl alcohol (25:24:1). We vortexed each mixture for 2 min and then centrifuged it for 5 min at 16,100 × *g* . After centrifugation, we removed the aqueous layer and mixed it with 2.5 volumes of 95% ethanol and 0.1 volume of 3 M sodium acetate (60); we incubated each aqueous layer mixture at –20°C for at least 3 h. Next, we centrifuged each aqueous layer mixture for 15 min at 16,100 × *g*, and removed the supernatant. Finally, we washed each pellet with 0.5 ml of 70% ethanol, centrifuged each mixture for 10 min at 16,100 × *g*, removed the supernatant, and resuspended each pellet in 50 μl of water.

We then assayed each DNA extract for the presence of each fungal taxon, or any fungal DNA in the case of the ITS1F/ITS4 primer pair, using PCR. Each PCR was composed of 7.9 μl of water, 0.1 μl of GoTaq Flexi polymerase (Promega), 5 μl of Flexi buffer with green dye added, 5 μl of 5× CES (combinatorial PCR enhancer solution [2.7 M betaine, 6.7 mM dithiothreitol, 6.7% dimethyl sulfoxide, 55 μg/ml bovine serum albumin]) (61), 5 μl of nucleotide mix, 2 μl of magnesium chloride, 1 μl of 10 μM concentrations of each primer, and 1 μl of undiluted template DNA extract. All reaction mixtures were cycled on a Bio-Rad iCycler or a myCycler using denaturing, annealing, and extension temperatures of 95, 55, and 72°C, respectively. We denatured the samples for 85 s and then ran 13 cycles of 35 s denaturing, 55 s annealing, and 45 s extension, followed by 13 cycles that were identical but had a 2-min extension, followed finally by 9 cycles with a 3-min extension. We ran a subsequent 10-min extension. Then, 2 μl of each PCR product was visualized on 1% agarose gels stained with SYBR Safe dye (Invitrogen) and photographed using a U:genius gel documenting system (Syngene) and a Stratagene transilluminator. Photographs of gels were scored for presence or absence of a band. Bands that were too faint to reliably score were run a second time with 6 μl of PCR product per well. The presence of a band on a gel indicated the presence of detectable fungal or yeast species DNA in a water sample.

To confirm that primers only amplified sequences from the target yeasts, we randomly selected nine PCR products generated from the C. pseudoglaebosa and R. babjevae primer pairs for sequencing. The primer pair that targets M. aphidis only amplified DNA from six pitcher water extracts, and we sequenced all six PCR products for this primer pair. Sequences were identical to or within one base of cultured isolate sequences.

### Pitcher water fungal DNA amplification and 454 sequencing.

We extracted and amplified fungal DNA for fungal community amplicon sequencing using the protocols described above, with the following changes. Gotaq Hotstart polymerase (Promega) was used instead of Flexi polymerase, and we used 50 μM instead of 10 μM the reverse primer. The forward primer consisted of (in order from 5′ to 3′) the 454 “A” primer (CCATCTCATCCCTGCGTGTCTCCGACTCAG) concatenated with a 10-bp multiplex tag (62) and ITS4; the reverse primer consisted of the 454 “B” primer (CCTATCCCCTGTGTGCCTTGGCAGTCTCAG) concatenated with ITS1F. Multiplex tags were unique to each sample. Reactions were cycled as follows: 95°C for 15 min; 30 cycles of 95°C for 1 min, 51°C for 1 min, and 72°C for 1 min; and a final extension of 72°C for 8 min.

Products were purified using Agencourt AMPure XP (Beckman Coulter) and quantitated using a Qubit dsDNA HS assay (Invitrogen) according to the manufacturers’ instructions. We combined equimolar concentrations of the products of each of three separate PCRs from each DNA extract. The sequencing pool consisted of pooled equimolar concentrations of each pooled PCR product. The pool was sequenced on one-eighth of a 454 Titanium sequencing run by the Duke Genome Sequencing and Analysis Core Resource.

### 454 sequence processing.

We processed sequences using QIIME 1.3.0 (63). Low-quality sequences were removed, and the remaining sequences were assigned to multiplex barcodes using the default quality filtering settings. Primers and barcodes were trimmed from each sequence, and sequences shorter than 200 bp and longer than 1,000 bp were removed from each data set. Sequences were denoised using the QIIME denoiser. We reduced chimeric sequences by trimming the 5.8s and ITS1 portions from all sequences using a fungal ITS extractor (64) and only analyzing the ITS2 portion. The 5.8s ribosomal region lies between the ITS1 and ITS2 spacers and is conserved among fungi relative to the spacers. We expected most chimeric sequences to form in the 5.8s region and to be composed of ITS1 and ITS2 sequences from different templates (65). We chose OTUs using the uclust method in QIIME, at 97% similarity (66). We discarded all OTUs composed of a single sequence (singleton OTUs) because we assumed that they resulted from sequencing errors. The longest sequence in each remaining cluster was retained as a representative sequence. Of the 141,424 total sequences produced, 27,632 were discarded for having lengths less than 200 bp or more than 1,000 bp, and 10,938 were discarded because they had low quality or did not have a matching barcode. Sixty-six sequences were discarded because the ITS2 subunit could not be extracted, and 139 sequences were discarded because they represented singleton OTUs. In total, we retained 102,649 sequences for further analysis. Each pitcher water sample produced between 253 and 4,365 sequences. Fastq files are available in the NCBI Sequence Read Archive (BioProject accession number PRJNA513075).

### Sequence taxonomy assignments.

We used the uclust method in QIIME and the UNITE database (dynamic release 01-12-2017) to assign taxonomy to a representative sequence for each OTU with a minimum percent similarity of 80% (66, 67). We assumed that unassigned OTUs were fungal sequences not yet in the UNITE database, and discarded one OTU assigned to the kingdom *Rhizaria*. We retained unassigned OTUs for diversity measurements but did not include them in taxonomy summaries. OTUs assigned to the genera *Candida*, *Rhodotorula*, and *Moesziomyces* were manually curated and compared to sequences for type strains in the NCBI database (68). In total, we detected 553 OTUs, of which 348 were assigned to fungal taxa, 1 was assigned to a kingdom other than *Fungi*, and 204 were not assigned. Of the 348 fungal taxa, 50% (174) were *Basidiomycota*, 47% (162) were *Ascomycota*, and 0.3% (12) were basal fungal lineages. Sequences, metadata, and OTU tables including taxonomic assignments in QIIME 1.3.0 format are available from the Edmond Open Access Data Repository (https://doi.org/10.17617/3.1w).

### Microcosm interaction assays.

Interactions between yeasts were assayed in microcosms designed to mimic pitchers simultaneously colonized by different numbers of two or more yeast species. Interacting yeasts grew in low-nutrient media designed to mimic natural conditions in pitchers. While sterilized pitcher liquid would be the most realistic media for microcosms, the quantities needed were unavailable. Instead, microcosms contained sterile yeast extract media (YEM) composed of 1 g/liter yeast extract in local tap water (Cambridge, MA). Tap water was used instead of deionized water because we wanted the media to include micronutrients present in local rainwater that may be important for pitcher plant yeast growth. The tap water supply in Cambridge, MA, where this experiment was conducted, comes from three Massachusetts reservoirs (69), and we expected it to have similar inputs as rainwater in Harvard Pond pitchers. Each microcosm was inoculated with a target yeast species and an interactor in 200 μl of liquid yeast media. Each target yeast was inoculated with about 1,000 cells per microcosm, and each interactor yeast was inoculated at zero, low, medium, and high cell numbers (0 and approximately 100, 1,000, and 10,000 cells).

Eighteen treatments of yeast mixtures were prepared, with ten replicates each, for a total of 180 microcosms. Before inoculation, yeasts were grown in liquid YEM for 48 h. Inoculation sizes were measured after inoculation using counts of CFU on solid YEM (YEM plus 1.5% agar). Microcosms were arranged in sterile 96-well polystyrene flat bottom cell culture plates and incubated between 25 and 27°C, with shaking at 700 rpm for 48 h. After incubation, 32 microcosms were discarded because of suspected contamination. We diluted each remaining microcosm 1:10^3^ or 1:10^4^ in sterile water, plated it to solid YEM, and counted CFU on plates containing at least 30 total CFU. The three species were distinguished by colony morphology (see above). When no CFU of an inoculated yeast were present on a plate, we conservatively assumed that the yeast was present in the microcosm in numbers just below our detection limit. We calculated total cell numbers assuming one instead of zero CFU for these yeasts absent from plates. CFU counts before and after incubation are available from the Edmond Open Access Data Repository (https://doi.org/10.17617/3.1w).

### Statistical analyses.

OTU data sets rarefied to 1,143 sequences were used to produce nonmetric multidimensional scaling (NMDS) plots and to compare community similarities and alpha diversity indices among pitchers. Eight samples contained fewer than 1,143 sequences and were discarded. Proportions of samples assigned to taxonomic groups were calculated based on the full nonrarefied data set. Community similarities over time were compared using partial distance-based redundancy analysis (db-RDA) of Jaccard dissimilarity (70) between each pair of samples with pitcher age as the explanatory variable, conditioned on pitcher identity. A correlation between geographic distance and community similarity was conducted using a partial Mantel test conditioned on pitcher age. Hill numbers of order *q* = 0 or 2 (^q^D) were calculated as ${}^{\text{q}}\text{D}={\left(\sum_{i=1}^{S}p_{i}^{q}\right)}^{1/(1-q)}$, where *S* is the total number of OTUs, and *p_i_* is the relative abundance of OTU *i*; ^1^D was calculated as the exponent of Shannon diversity (37, 38). Changes in Hill numbers were modeled over time using repeated-measures linear models controlled for pitcher identity; ^1^D and ^2^D were log transformed before analyses to homogenize variances among time points, and ^0^D was not transformed.

We modeled the impact of interactor yeasts on focal yeasts in microcosms using multiple linear and polynomial regressions. Separate regressions were conducted for each focal yeast. For each regression, focal yeast yield was the dependent variable, and both the number of coinoculated interactor yeast cells and the identity of the interactor yeast were independent variables. We modeled both linear and quadratic relationships between the number of coinoculated interactor yeast cells and the dependent variable because the relationship did not always appear linear when plotted. Before constructing the regressions, we square-root-transformed focal yeast yield to homogenize variances for the focal yeasts R. babjevae and C. pseudoglaebosa, but left yield untransformed for the focal yeast M. aphidis. We also transformed competitor inoculum size by log_10_(*x* + 1) because interactor inoculum size was varied on a log scale in the experiment. When comparing the influences of competitor species, we randomly assigned treatments with no interacting yeast inoculum to one of the two interacting yeasts. When selecting the best-fitting regression model, we first established the best-fitting relationship (linear, quadratic, or both) between log-transformed interactor inoculum size and focal yeast yield and then determined whether adding interactor identity or interactions between interacting yeast identity and inoculum size to the model improved it. The best-fitting model was the one with the lowest Akaike Information Criterion (AIC).

All statistical analyses and index calculations were conducted using R version 3.3.1 (71) and the packages vegan, fields, nlme, and GUniFrac (72–75). Plots were made using ggplot2 (76).

### Data availability.

Fastq files have been deposited in the NCBI Sequence Read Archive database (see BioProject accession number PRJNA513075). Representative sequences for each OTU, metadata, OTU tables (including taxonomic assignments in QIIME 1.3.0 format), and raw data from the competition experiment have been deposited the Edmond Open Access Data Repository (https://doi.org/10.17617/3.1w).

## References

[1] Fierer N, Nemergut D, Knight R, Craine JM 2010 Changes through time: integrating microorganisms into the study of succession. Res Microbiol 161:635–642. doi:10.1016/j.resmic.2010.06.002. 20599610

[2] Boynton PJ, Greig D 2016 Species richness influences wine ecosystem function through a dominant species. Fungal Ecol 22:61–72. doi:10.1016/j.funeco.2016.04.008.

[3] Copeland JK, Yuan L, Layeghifard M, Wang PW, Guttman DS 2015 Seasonal community succession of the phyllosphere microbiome. Mol Plant-Microbe Interact 28:274–285. doi:10.1094/MPMI-10-14-0331-FI. 25679538

[4] Cutler NA, Chaput DL, van der Gast CJ 2014 Long-term changes in soil microbial communities during primary succession. Soil Biol Biochem 69:359–370. doi:10.1016/j.soilbio.2013.11.022.

[5] Koenig JE, Spor A, Scalfone N, Fricker AD, Stombaugh J, Knight R, Angenent LT, Ley RE 2011 Succession of microbial consortia in the developing infant gut microbiome. Proc Natl Acad Sci U S A 108:4578–4585. doi:10.1073/pnas.1000081107. 20668239 PMC3063592

[6] Redford AJ, Fierer N 2009 Bacterial succession on the leaf surface: a novel system for studying successional dynamics. Microb Ecol 58:189–198. doi:10.1007/s00248-009-9495-y. 19221834

[7] Zumsteg A, Luster J, Goransson H, Smittenberg RH, Brunner I, Bernasconi SM, Zeyer J, Frey B 2012 Bacterial, archaeal and fungal succession in the forefield of a receding glacier. Microb Ecol 63:552–564. doi:10.1007/s00248-011-9991-8. 22159526

[8] Wolfe BE, Button JE, Santarelli M, Dutton RJ 2014 Cheese rind communities provide tractable systems for in situ and *in vitro* studies of microbial diversity. Cell 158:422–433. doi:10.1016/j.cell.2014.05.041. 25036636 PMC4222527

[9] Findley K, Oh J, Yang J, Conlan S, Deming C, Meyer JA, Schoenfeld D, Nomicos E, Park M, Kong HH, Segre JA 2013 Topographic diversity of fungal and bacterial communities in human skin. Nature 498:367–370. doi:10.1038/nature12171. 23698366 PMC3711185

[10] Jiao S, Chen W, Wang E, Wang J, Liu Z, Li Y, Wei G 2016 Microbial succession in response to pollutants in batch-enrichment culture. Sci Rep 6:21791. doi:10.1038/srep21791. 26905741 PMC4764846

[11] Hillebrand H, Bennett DM, Cadotte MW 2008 Consequences of dominance: a review of evenness effects on local and regional ecosystem processes. Ecology 89:1510–1520. 18589516 10.1890/07-1053.1

[12] Albergaria H, Francisco D, Gori K, Arneborg N, Girio F 2010 *Saccharomyces cerevisiae* CCMI 885 secretes peptides that inhibit the growth of some non-*Saccharomyces* wine-related strains. Appl Microbiol Biotechnol 86:965–972. doi:10.1007/s00253-009-2409-6. 20039034

[13] Goddard MR 2008 Quantifying the complexities of *Saccharomyces cerevisiae*’s ecosystem engineering via fermentation. Ecology 89:2077–2082. 18724717 10.1890/07-2060.1

[14] Nissen P, Arneborg N 2003 Characterization of early deaths of non-*Saccharomyces* yeasts in mixed cultures with *Saccharomyces cerevisiae*. Arch Microbiol 180:257–263. doi:10.1007/s00203-003-0585-9. 12898132

[15] Williams KM, Liu P, Fay JC 2015 Evolution of ecological dominance of yeast species in high-sugar environments. Evolution 69:2079–2093. doi:10.1111/evo.12707. 26087012 PMC4751874

[16] Ganucci D, Guerrini S, Mangani S, Vincenzini M, Granchi L 2018 Quantifying the effects of ethanol and temperature on the fitness advantage of predominant *Saccharomyces cerevisiae* strains occurring in spontaneous wine fermentations. Front Microbiol 9:1563. doi:10.3389/fmicb.2018.01563. 30057578 PMC6053494

[17] Khan FA, Ansari AA 2005 Eutrophication: an ecological vision. Botanical Rev 71:449–482. doi:10.1663/0006-8101(2005)071[0449:EAEV]2.0.CO;2.

[18] Wolfe BE, Dutton RJ 2015 Fermented foods as experimentally tractable microbial ecosystems. Cell 161:49–55. doi:10.1016/j.cell.2015.02.034. 25815984

[19] Fukami T 2015 Historical contingency in community assembly: integrating niches, species pools, and priority effects. Annu Rev Ecol Evol Syst 46:1–23. doi:10.1146/annurev-ecolsys-110411-160340.

[20] Wilson MJ, McTammany ME 2016 Spatial scale and dispersal influence metacommunity dynamics of benthic invertebrates in a large river. Freshwater Science 35:738–747. doi:10.1086/685732.

[21] Holyoak M, Leibold MA, Mouquet N, Holt RD, Hoopes MF 2005 Metacommunities: a framework for large-scale community ecology, p 1–31. *In* Holyoak M, Leibold MA, Holt RD (ed), Metacommunities: spatial dynamics and ecological communities. The University of Chicago Press, Chicago, IL.

[22] Logue JB, Mouquet N, Peter H, Hillebrand H 2011 Empirical approaches to metacommunities: a review and comparison with theory. Trends Ecol Evol 26:482–491. doi:10.1016/j.tree.2011.04.009. 21641673

[23] Leibold MA, Holyoak M, Mouquet N, Amarasekare P, Chase JM, Hoopes MF, Holt RD, Shurin JB, Law R, Tilman D, Loreau M, Gonzalez A 2004 The metacommunity concept: a framework for multi-scale community ecology. Ecol Lett 7:601–613. doi:10.1111/j.1461-0248.2004.00608.x.

[24] Mouquet N, Munguia P, Kneitel JM, Miller TE 2003 Community assembly time and the relationship between local and regional species richness. Oikos 103:618–626. doi:10.1034/j.1600-0706.2003.12772.x.

[25] Sferra CO, Hart JL, Howeth JG 2017 Habitat age influences metacommunity assembly and species richness in successional pond ecosystems. Ecosphere 8:e01871. doi:10.1002/ecs2.1871.

[26] Buckley HL, Miller TE, Ellison AM, Gotelli NJ 2003 Reverse latitudinal trends in species richness of pitcher-plant food webs. Ecol Lett 6:825–829. doi:10.1046/j.1461-0248.2003.00504.x.

[27] Peterson CN, Day S, Wolfe BE, Ellison AM, Kolter R, Pringle A 2008 A keystone predator controls bacterial diversity in the pitcher-plant (*Sarracenia purpurea*) microecosystem. Environ Microbiol 10:2257–2266. doi:10.1111/j.1462-2920.2008.01648.x. 18479443

[28] Bennett KF, Ellison AM 2009 Nectar, not colour, may lure insects to their death. Biol Lett 5:469–472. doi:10.1098/rsbl.2009.0161. 19429649 PMC2781919

[29] Ellison AM, Gotelli NJ, Brewer JS, Cochran-Stafira DL, Kneitel JM, Miller TE, Worley AC, Zamora R 2003 The evolutionary ecology of carnivorous plants. Adv Ecol Res 33:1–74.

[30] Cochran-Stafira DL, von Ende CN 1998 Integrating bacteria into food webs: studies with *Sarracenia purpurea* inquilines. Ecology 79:880–898. doi:10.2307/176587.

[31] Bittleston LS, Wolock CJ, Yahya BE, Chan XY, Chan KG, Pierce NE, Pringle A 2018 Convergence between the microcosms of Southeast Asian and North American pitcher plants. Elife 7:e37641. 30152327 10.7554/eLife.36741 PMC6130972

[32] Judd WW 1959 Studies of the Byron Bog in southwestern Ontario. X. Inquilines and victims of the pitcher-plant, *Sarracenia purpurea* L. Can Entomol 91:171–180. doi:10.4039/Ent91171-3.

[33] Atwater DZ, Butler JL, Ellison AM 2006 Spatial distribution and impacts of moth herbivory on northern pitcher plants. Northeastern Naturalist 13:43–56. doi:10.1656/1092-6194(2006)13[43:SDAIOM]2.0.CO;2.

[34] Gardes M, Bruns TD 1993 ITS primers with enhanced specificity for basidiomycetes–application to the identification of mycorrhizae and rusts. Mol Ecol 2:113–118. 8180733 10.1111/j.1365-294x.1993.tb00005.x

[35] White TJ, Bruns T, Lee S, Taylor J 1990 Amplification and direct sequencing of fungal ribosomal RNA genes for phylogenetics, p 315–322. *In* Innis MA, Gelfand DH, Sninsky JJ, White TJ (ed), PCR protocols: a guide to methods and applications. Academic Press, Inc, New York, NY.

[36] Schoch CL, Seifert KA, Huhndorf S, Robert V, Spouge JL, Levesque CA, Chen W 2012 Nuclear ribosomal internal transcribed spacer (ITS) region as a universal DNA barcode marker for Fungi. Proc Natl Acad Sci U S A 109:6241–6246. doi:10.1073/pnas.1117018109. 22454494 PMC3341068

[37] Chao A, Gotelli NJ, Hsieh TC, Sander EL, Ma KH, Colwell RK, Ellison AM 2014 Rarefaction and extrapolation with Hill numbers: a framework for sampling and estimation in species diversity studies. Ecol Monogr 84:45–67. doi:10.1890/13-0133.1.

[38] Hill MO 1973 Diversity and evenness: a unifying notation and its consequences. Ecology 54:427–432. doi:10.2307/1934352.

[39] Goldberg DE, Werner PA 1983 Equivalence of competitors in plant communities: a null hypothesis and a field experimental approach. Am J Bot 70:1098–1104. doi:10.1002/j.1537-2197.1983.tb07912.x.

[40] Boynton PJ 2012 Ecological patterns and processes in Sarracenia carnivorous pitcher plant fungi. PhD thesis Harvard University, Cambridge, MA.

[41] Scheffer M, van Bavel B, van de Leemput IA, van Nes EH 2017 Inequality in nature and society. Proc Natl Acad Sci U S A 114:13154–13157. doi:10.1073/pnas.1706412114. 29183971 PMC5740652

[42] Starmer WT, Lachance MA 2011 Yeast ecology, p 65–86. *In* Kurtzman CP, Fell JW, Boekhout T (ed), The yeasts: a taxonomic study. Elsevier, Atlanta, GA.

[43] Reeves WK 2004 Oviposition by *Aedes aegypti* (Diptera: Culicidae) in relation to conspecific larvae infected with internal symbiotes. J Vector Ecol 29:159–163. 15266753

[44] Gilpin ME, Carpenter P, Pomerantz MJ 1986 The assembly of a laboratory community: multispecies competition in *Drosophila*, p 23–40. *In* Diamond J, Case TJ (ed), Community ecology. Harper & Row, New York, NY.

[45] Miller TE, Kneitel JM 2005 Inquiline communities in pitcher plants as a prototypical metacommunity, p 122–145. *In* Holyoak M, Leibold MA, Holt RD (ed), Metacommunities: spatial dynamics and ecological communities. The University of Chicago Press, Chicago, IL.

[46] Fish D, Hall DW 1978 Succession and stratification of aquatic insects inhabiting the leaves of the insectivorous pitcher plant, *Sarracenia purpurea*. Am Midl Nat 99:172–183. doi:10.2307/2424941.

[47] Gray SM, Akob DM, Green SJ, Kostka JE 2012 The bacterial composition within the *Sarracenia purpurea* model system: local scale differences and the relationship with the other members of the food web. PLoS One 7:e50969. doi:10.1371/journal.pone.0050969. 23227224 PMC3515446

[48] Miller TE, terHorst CP 2012 Testing successional hypotheses of stability, heterogeneity, and diversity in pitcher-plant inquiline communities. Oecologia 170:243–251. doi:10.1007/s00442-012-2292-1. 22430372

[49] Connell JH, Slatyer RO 1977 Mechanisms of succession in natural communities and their role in community stability and organization. Am Nat 111:1119–1144. doi:10.1086/283241.

[50] Cooper WS 1923 The recent ecological history of Glacier Bay, Alaska. II. The present vegetation cycle. Ecology 4:223–246. doi:10.2307/1929047.

[51] Hiscox J, Boddy L 2017 Armed and dangerous: chemical warfare in wood decay communities. Fungal Biol Rev 31:169–184. doi:10.1016/j.fbr.2017.07.001.

[52] Holmer L, Stenlid J 1997 Competitive hierarchies of wood decomposing *Basidiomycetes* in artificial systems based on variable inoculum sizes. Oikos 79:77–84. doi:10.2307/3546092.

[53] Swan JMA, Gill AM 1970 The origins, spread, and consolidation of a floating bog in Harvard Pond, Petersham, Massachusetts. Ecology 51:829–840. doi:10.2307/1933975.

[54] Ellison AM, Farnsworth EJ, Gotelli NJ 2002 Ant diversity in pitcher-plant bogs of Massachusetts. Northeastern Nat 9:267–284. doi:10.1656/1092-6194(2002)009[0267:ADIPPB]2.0.CO;2.

[55] Hausner G, Reid J, Klassen G 1993 On the subdivision of *Ceratocystis* SL based on partial ribosomal DNA-sequences. Can J Bot 71:52–63. doi:10.1139/b93-007.

[56] Vilgalys R, Hester M 1990 Rapid genetic identification and mapping of enzymatically amplified ribosomal DNA from several *Cryptococcus* species. J Bacteriol 172:4238–4246. 2376561 10.1128/jb.172.8.4238-4246.1990 PMC213247

[57] Zhang Z, Schwartz S, Wagner L, Miller W 2000 A greedy algorithm for aligning DNA sequences. J Comput Biol 7:203–214. doi:10.1089/10665270050081478. 10890397

[58] Rozen S, Skaletsky H 1999 Primer3 on the WWW for general users and for biologist programmers, p 365–386. *In* Misener S, Krawetz SA (ed), Bioinformatics methods and protocols. Humana Press, Totowa, NJ. 10.1385/1-59259-192-2:365 10547847

[59] Hoffman CS 1997 Preparations of yeast DNA, RNA, and proteins. Curr Protoc Cell Bio 39:13.11.1–13.11.4. doi:10.1002/0471142727.mb1311s39.

[60] Sambrook J, Russell DW 2001 Molecular cloning: a laboratory manual, 3rd ed Cold Spring Harbor Laboratory Press, Cold Spring Harbor, NY.

[61] Ralser M, Querfurth R, Warnatz HJ, Lehrach H, Yaspo ML, Krobitsch S 2006 An efficient and economic enhancer mix for PCR. Biochem Biophys Res Commun 347:747–751. doi:10.1016/j.bbrc.2006.06.151. 16842759

[62] 454 Live Sciences Corporation. 2009 Using multiplex identifier (MID) adaptors for the GS FLX Titanium Chemistry—extended MID set *In* Genome Sequencer FLX System Technical Bulletin No. 005-2009. Roche Applied Science, Basel, Switzerland.

[63] Caporaso JG, Kuczynski J, Stombaugh J, Bittinger K, Bushman FD, Costello EK, Fierer N, Pena AG, Goodrich JK, Gordon JI, Huttley GA, Kelley ST, Knights D, Koenig JE, Ley RE, Lozupone CA, McDonald D, Muegge BD, Pirrung M, Reeder J, Sevinsky JR, Turnbaugh PJ, Walters WA, Widmann J, Yatsunenko T, Zaneveld J, Knight R 2010 QIIME allows analysis of high-throughput community sequencing data. Nat Methods 7:335–336. doi:10.1038/nmeth.f.303. 20383131 PMC3156573

[64] Nilsson RH, Veldre V, Hartmann M, Unterseher M, Amend A, Bergsten J, Kristiansson E, Ryberg M, Jumpponen A, Abarenkov K 2010 An open source software package for automated extraction of ITS1 and ITS2 from fungal ITS sequences for use in high- throughput community assays and molecular ecology. Fungal Ecol 3:284–287. doi:10.1016/j.funeco.2010.05.002.

[65] Nilsson RH, Abarenkov K, Veldre V, Nylinder S, De Wit P, Brosché S, Alfredsson JF, Ryberg M, Kristiansson E 2010 An open source chimera checker for the fungal ITS region. Mol Ecol Resour 10:1076–1081. doi:10.1111/j.1755-0998.2010.02850.x. 21565119

[66] Edgar RC 2010 Search and clustering orders of magnitude faster than BLAST. Bioinformatics 26:2460–2461. doi:10.1093/bioinformatics/btq461. 20709691

[67] Nilsson RH, Larsson KH, Taylor AFS, Bengtsson-Palme J, Jeppesen TS, Schigel D, Kennedy P, Picard K, Glockner FO, Tedersoo L, Saar I, Koljalg U, Abarenkov K 2018 The UNITE database for molecular identification of fungi: handling dark taxa and parallel taxonomic classifications. Nucleic Acids Res doi:10.1093/nar/gky1022. PMC6324048 30371820

[68] Benson DA, Karsch-Mizrachi I, Lipman DJ, Ostell J, Wheeler DL 2005 GenBank. Nucleic Acids Res 33:D34–D38. doi:10.1093/nar/gki063. 15608212 PMC540017

[69] Waldron MC, Bent GC 2001 Factors affecting reservoir and stream-water quality in the Cambridge, Massachusetts, drinking-water source area and implications for source-water protection, U.S. Department of the Interior, U.S. Geological Survey. Water-Resources Investigations Report 2001:4262.

[70] Jaccard P 1912 The distribution of the flora in the alpine zone. New Phytol 11:37–50. doi:10.1111/j.1469-8137.1912.tb05611.x.

[71] R Development Core Team. 2016 R: a language and environment for statistical computing. R Foundation for Statistical Computing, Vienna, Austria.

[72] Chen J 2018 GUniFrac: Generalized UniFrac Distances, vR package version 1.1. https://CRAN.R-project.org/package=GUniFrac.

[73] Nychka D, Furrer R, Paige J, Sain S 2016 fields: tools for spatial data, vR package version 9.0. www.image.ucar.edu/fields.

[74] Oksanen J, Blanchet FG, Friendly M, Kindt R, Legendre P, McGlinn D, Minchin PR, O’Hara RB, Simpson GL, Solymos P, Stevens MHH, Szoecs E, Wagner H 2016 vegan: Community Ecology Package, vR package version 2.4-1. https://CRAN.R-project.org/package=vegan.

[75] Pinheiro J, Bates D, DebRoy S, Sarkar D, Eispack A, Heisterkamp S, Van Willigen B, R Development Core Team 2016 nlme: Linear and Nonlinear Mixed Effects Models, vR package version 3.1-128. http://CRAN.R-project.org/package=nlme.

[76] Wickham H 2016 ggplot2: elegant graphics for data analysis. Springer, New York, NY.

[77] Environmental Systems Resource Institute. 2006 Arcmap, v9.2. ESRI, New York, NY.

[78] Office of Geographic Information (MassGIS). 2000 1:5,000 black and white digital orthophoto images. Commonwealth of Massachusetts Information Technology Division, Boston, MA.

[79] National Atlas of the United States. 2006 State boundaries of the United States. http://www.nationalatlas.gov/.

